# Trunk rotation coordination in front crawl: which segment leads? Three coordination strategies and their dynamic changes across 400-m split progression in elite swimmers

**DOI:** 10.3389/fbioe.2026.1857371

**Published:** 2026-07-23

**Authors:** Yiming Niu, Lihang Jiang, Mingyu Shang, Jie Gao

**Affiliations:** 1 Beijing Sport University, Beijing, China; 2 Tsinghua University High School, Beijing, China

**Keywords:** Bayesian multilevel model, Biomechanics, body roll, motion analysis, motor coordination, wearable inertial sensors

## Abstract

**Objective:**

To identify upper-trunk–pelvic roll coordination strategies during 400-m front-crawl swimming and examine their kinematic characteristics, segment-wise distribution, and associations with swimming speed.

**Methods:**

Twenty-two elite male swimmers completed a maximal 400-m front-crawl trial while two inertial measurement units recorded upper-trunk and pelvic roll. Cycle-level cross-correlation lag was used to classify coordination into upper-trunk-leading (UL), pelvic-leading (PL), and near-synchronous (NS) strategies. Kinematic differences among strategies and changes in strategy probability across 50-m segments and swimming speed were examined using Bayesian multilevel models.

**Results:**

Across 2,796 valid roll cycles, NS was the most frequent strategy (53.04%), followed by PL (25.86%) and UL (21.10%). Strategy use varied substantially between swimmers. UL showed greater torso twist range than NS (NS–UL = −0.71, 95% CrI: −0.96 to −0.42) and greater torso twist velocity than both NS (NS–UL = −0.61, 95% CrI: −0.95 to −0.26) and PL (UL–PL = 0.62, 95% CrI: 0.19–1.04). In contrast, upper-trunk and pelvic roll ranges were larger in NS and PL than in UL, with no clear difference between NS and PL. Faster within-athlete speed increased the odds of UL relative to both NS (OR = 2.38, 95% CrI: 1.76–3.26) and PL (OR = 2.07, 95% CrI: 1.31–3.33). No clear segment effects or between-athlete mean speed effects were observed.

**Conclusion:**

Elite 400-m front-crawl swimmers used three distinct upper-trunk–pelvic lead–lag strategies, with marked inter-individual variability. NS was most common, whereas UL was more likely at faster within-athlete speeds. These findings suggest that trunk coordination in elite swimmers is individualized rather than organized around a single optimal pattern.

## 1 Introduction

In front-crawl swimming, body roll refers to the rotational movement of the trunk about its longitudinal axis (Counsilman, 1968). The magnitude of body roll varies according to swimming speed, exercise intensity, and breathing pattern (Payton et al., 1999; Castro et al., 2006). However, this definition treats the trunk as a single rigid segment. To provide a more detailed description of trunk rotation, subsequent studies have partitioned body roll into shoulder roll and hip roll, which represent the rotational motions of the upper and lower trunk segments, respectively, about the longitudinal axis of the body (Cappaert et al., 1995; Psycharakis and Sanders, 2008; Yanai, 2001). Whether considered as a whole-body movement or as segment-specific rotations, body roll, including both shoulder roll and hip roll, plays an important role in swimming technique, propulsion, and performance (Psycharakis and Ross, 2010; Liu et al., 1993; Kudo et al., 2019).

Although shoulder roll and hip roll both exhibit approximately sinusoidal patterns throughout the stroke cycle, their spatiotemporal characteristics are not identical and are influenced by swimming speed. Previous studies comparing sprint and distance specialists at the same swimming pace (sprint pace or 400-m pace) found only small differences in shoulder and hip roll, suggesting that roll characteristics may depend more on swimming pace than on event specialization (McCabe et al., 2011; McCabe and Ross, 2012). Studies comparing sprint and distance-paced swimming have reported that the amplitudes of both shoulder roll and hip roll decrease as swimming speed increases (Gonjo et al., 2021; Andersen et al., 2020). Furthermore, shoulder roll amplitude is consistently greater than hip roll amplitude across a range of swimming speeds (Cappaert et al., 1995; Yanai, 2003; Psycharakis and Sanders, 2008; McCabe et al., 2011; McCabe and Ross, 2012). The reduction in shoulder roll amplitude at higher speeds may be attributable to the inverse relationship between roll amplitude and stroke frequency (Yanai, 2003). In contrast, changes in hip roll may be influenced by kicking activity, as kicking has been shown to suppress rotational motion of the hips (Sanders and Psycharakis, 2009). A study of 200-m front-crawl swimming further demonstrated that faster swimmers exhibited smaller shoulder roll amplitudes than slower swimmers and that hip roll amplitude increased progressively throughout the race as fatigue developed, potentially owing to a reduction in compensatory lower-limb contributions (Psycharakis and Sanders, 2008). More recently, a study of technical fatigue during a 50-m front-crawl swim in youth swimmers reported a significant increase in hip roll amplitude during the second 25 m compared with the first 25 m (Skorulski et al., 2025). Collectively, these findings suggest that shoulder and hip roll characteristics are influenced not only by swimming speed but also by fatigue-related changes occurring during prolonged swimming.

The relative angular difference between shoulder roll and hip roll indicates the presence of twist within the torso (Psycharakis and Sanders, 2008; Psycharakis and McCabe, 2011; McCabe and Ross, 2012). This shoulder–hip angular difference was subsequently operationally quantified as torso twist, which also appears to be strongly influenced by swimming speed (Andersen et al., 2020). Some studies have reported that increasing swimming speed leads to a marked reduction in hip roll amplitude while shoulder roll amplitude remains relatively unchanged (Andersen et al., 2020). In contrast, other studies have observed a larger reduction in shoulder roll amplitude than in hip roll amplitude under faster swimming conditions (Psycharakis and Sanders, 2008; Hyodo et al., 2023). These findings indicate that although torso twist is consistently observed during front-crawl swimming, the segment primarily responsible for this phenomenon and its underlying mechanisms remain unclear. A recent flume-based study conducted under a 10-s sprint condition suggested that a greater torso twist angle may contribute to improved swimming velocity (Hyodo et al., 2023). One potential explanation is that increased torso twist reduces hydrodynamic energy losses in non-propulsive directions (Yanai, 2003). Consequently, swimmers may enhance performance by generating and controlling relative longitudinal rotation between the upper and lower trunk segments (Figueiredo et al., 2013; Psycharakis and Ross, 2010; Andersen et al., 2023). However, the calculation of torso twist has traditionally focused on spatial differences between shoulder roll and hip roll (Andersen et al., 2020; Andersen et al., 2023; Hyodo et al., 2023), whereas comparatively little attention has been paid to their temporal coordination. Previous work has suggested that temporal offsets between shoulder roll and hip roll may exist among swimmers (Psycharakis and Sanders, 2008). Such dissociations between spatial and temporal characteristics imply a degree of independence between upper- and lower-trunk rotational movements (Andersen et al., 2023). Because similar torso twist magnitudes may arise from different shoulder–hip timing patterns, analysis of spatial differences alone may be insufficient to fully characterize trunk coordination.

The timing of body roll and the sequence of segmental movements during front-crawl swimming remain subjects of debate (Lee et al., 2008). One view proposes that body roll follows arm movement (Maglischo, 2003), whereas another suggests that hip rotation precedes arm motion (Prichard, 1993). Previous investigations of shoulder–hip timing relationships have predominantly employed discrete-event approaches, in which the timing of specific events, such as roll onset, peak roll, or roll termination, is compared between the shoulder and hip segments. Early work examining rotational timing reported that sub-elite swimmers exhibited anti-phase shoulder–hip rolling patterns during a 100-m race, whereas elite swimmers demonstrated in-phase rolling patterns (Cappaert et al., 1995)). Using the timing of peak roll and neutral positions, Psycharakis reported substantial inter-individual variability during 200-m front-crawl swimming, with both shoulder-leading and hip-leading patterns observed (Psycharakis and Sanders, 2008). Similar inter-individual variability was subsequently reported during 25-m sprint front-crawl swimming (McCabe et al., 2011). A recent study comparing para-swimmers and non-disabled swimmers examined the timing of roll termination and reported that hip roll generally ended before shoulder roll, although between-group differences were not significant and substantial inter-individual variability remained (Lee et al., 2025). Other studies have reported the timing of shoulder and hip roll peaks but focused primarily on comparisons between populations or pace conditions rather than direct evaluation of shoulder–hip timing relationships (Gonjo et al., 2021; Hyodo et al., 2023; McCabe et al., 2011; McCabe and Ross, 2012). Overall, discrete-event studies have revealed considerable variability in shoulder–hip timing relationships, suggesting that these relationships may be influenced by swimming speed, individual technique characteristics, and methodological differences.

A smaller number of studies have adopted continuous-signal approaches to examine the temporal relationship between complete shoulder and hip roll waveforms. Using Fourier analysis under submaximal swimming conditions, Yanai reported that hip roll lagged shoulder roll by approximately 1% of the stroke cycle and concluded that shoulder and hip roll motions were nearly synchronized (Yanai, 2001). Using a similar Fourier-based approach during 200-m front-crawl swimming, Sanders reported relatively small phase differences between shoulder and hip roll, together with substantial inter-individual variability, including both shoulder-leading and hip-leading patterns (Sanders and Psycharakis, 2009). More recently, Nikodelis applied a cross-correlation lag method during 25-m sprint swimming and observed a consistent hip-leading pattern across all participants (Nikodelis et al., 2023). These findings indicate that the nature of shoulder–hip phase relationships during front-crawl swimming remains unclear. In particular, little is known about whether these relationships change in response to fatigue-related technical adjustments during prolonged swimming under different distance and pace conditions.

From the perspective of dynamical systems theory, elucidating coordination mechanisms requires the analysis of a large number of movement trajectories to reveal the underlying coordination patterns, while also considering the stability of these patterns over time (Stergiou, 2004). Most previous studies have focused on sprint swimming or have simulated distance-paced conditions using short-duration trials. Even studies involving continuous swimming typically analyzed only a single stroke cycle within each race segment (Cappaert et al., 1995; Psycharakis and Sanders, 2008; Sanders and Psycharakis, 2009; McCabe et al., 2011; McCabe and Ross, 2012). With the development of microelectromechanical systems technology, inertial measurement units have become powerful tools for human movement analysis (Yang and Li, 2012). Owing to their small size, wearability, and ease of deployment (Hamidi Rad et al., 2021; James et al., 2011), inertial measurement units enable continuous monitoring over extended periods and across multiple stroke cycles in ecologically valid swimming environments. Furthermore, their measurements have been shown to be comparable with those obtained using video-based and other labor-intensive methods (Stadnik et al., 2004). In studies of swimming trunk coordination, inertial measurement units are commonly placed on the upper-trunk and pelvic to characterize the movement rhythms of different trunk segments (Bächlin and Tröster, 2012; Averianova et al., 2016; Nikodelis et al., 2023; Nikodelis et al., 2013). Compared with analyses based on a limited number of stroke cycles, the examination of large numbers of consecutive cycles may provide a more comprehensive representation of intersegmental coordination because it captures a broader range of movement variability and coordination behaviors (Nikodelis et al., 2023).

At present, there is a lack of research systematically examining upper-trunk and pelvic roll timing relationships and their evolution throughout prolonged swimming using large datasets of continuous stroke cycles. The 400-m front-crawl event occupies a unique position near the transition between aerobic and anaerobic energy supply systems and requires complex pacing decisions. It is frequently used to evaluate swimming kinematics, movement coordination, swimming efficiency, and energetic characteristics (Correia et al., 2023). Therefore, the present study selected the 400-m front-crawl event as the experimental task. Compared with shorter-distance events, the 400-m event combines prolonged duration with substantial metabolic demands, making it more likely to reveal fatigue-related adjustments in technique and coordination during continuous swimming. Consequently, it provides an appropriate context for examining changes in upper-trunk and pelvic roll timing relationships across different phases of a race.

The aim of the present study was to investigate upper-trunk and pelvic roll timing relationships in elite swimmers during a 400-m front-crawl swim and to further examine whether these coordination patterns change across race segments and swimming speeds, together with their associated kinematic characteristics.

## 2 Methods

### 2.1 Participant

An *a priori* power analysis was conducted using G*Power 3.1.9.7 (Faul et al., 2007). For a repeated-measures design, we assumed a medium effect size (f = 0.25), a significance level of α = 0.05, and a desired statistical power of (1−β) = 0.95. The analysis indicated a minimum required sample size of 18 participants. Guided by McKay’s athlete classification framework (McKay et al., 2022), we recruited 22 male swimmers, including eight international-level athletes (age: 18.88 ± 1.64 years; height: 184.13 ± 2.85 cm; body mass: 77.5 ± 9.53 kg; training experience: 11.0 ± 1.85 years; 400 m front-crawl personal best: 236.40 ± 2.14 s) and 14 national-level athletes (age: 18.00 ± 2.04 years; height: 180.64 ± 6.11 cm; body mass: 74.36 ± 9.12 kg; training experience: 9.71 ± 1.44 years; 400 m front-crawl personal best: 257.00 ± 6.61 s). Participants were drawn from all eligible athletes within the administrative region of Beijing, China, who met the prespecified criteria for training experience and performance level.

Inclusion criteria were: (i) front crawl as the primary competitive stroke; (ii) right-side breathing to maintain consistency in breathing laterality across participants; and (iii) at least 5 years of systematic training. Swimmers were excluded if they had sustained an injury within the preceding 6 months that prevented participation in testing. The study was approved by the Sports Science Experiment Ethics Committee of Beijing Sport University (2025342H), and written informed consent was obtained from all participants and their legal guardians.

### 2.2 Data capture and processing

Two IMUs (MTi-3; Beijing Aerospace Era Optoelectronic Technology Co., Ltd., Beijing, China) were used to synchronously capture upper-trunk- and pelvic-roll kinematics during front crawl. Each IMU measured 29 × 45.5 × 8.5 mm and sampled at 100 Hz, with a manufacturer-reported angular velocity range of 0 to ±500°·s^-1^ and a manufacturer-reported angular measurement error of <1.0°.

Mote: R/L shoulder, right/left acromion; R/L PSIS, right/left posterior superior iliac spine. Y-axis, cranio-caudal direction; X-axis, mediolateral direction; Z-axis, anteroposterior direction.

**FIGURE 1:**
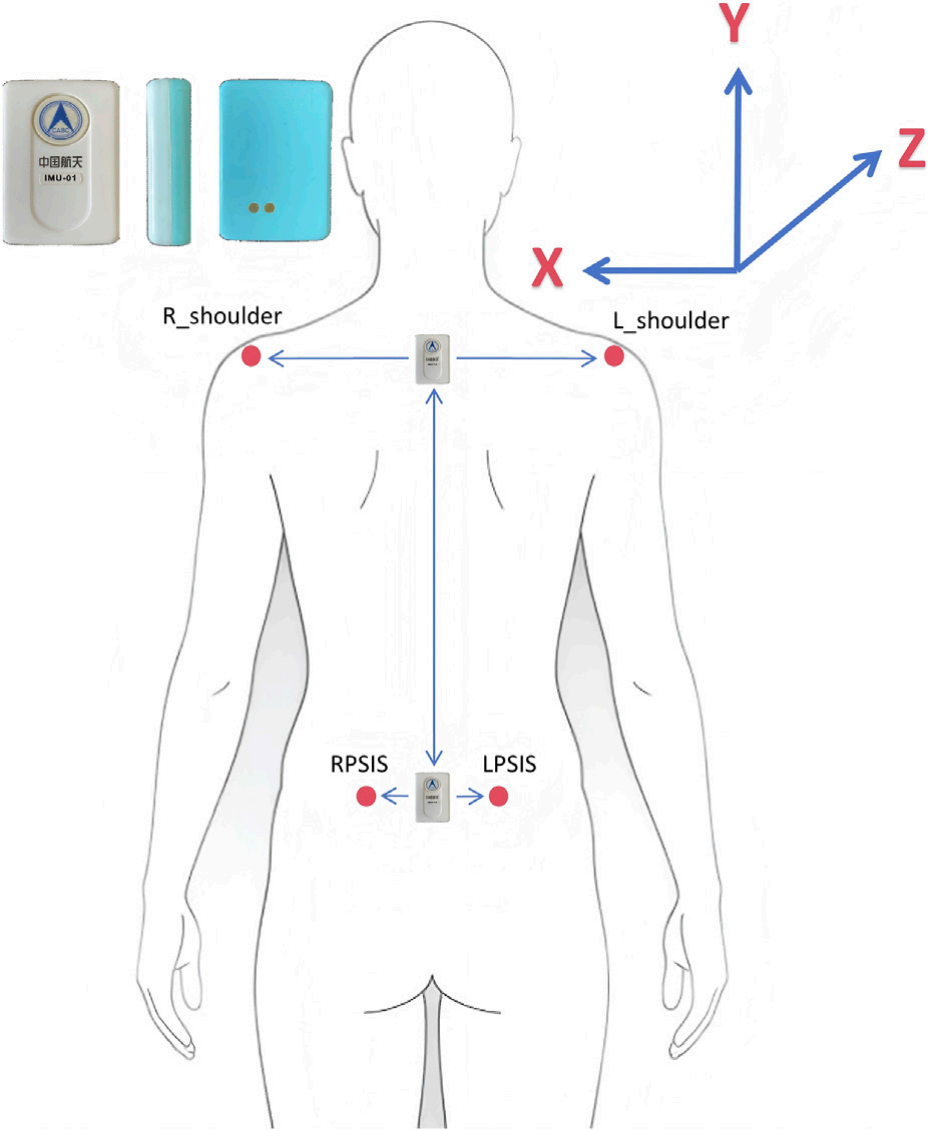
Schematic of IMU placement and the trunk coordinate system.

As shown in Figure 1, the upper-trunk IMU was secured at the midpoint of the line connecting the left and right acromion processes (Crenna et al., 2025), whereas the pelvic IMU was secured over the sacral region, at the midpoint between the left and right posterior superior iliac spines (PSIS) (Hamidi Rad et al., 2021). Both sensors were affixed with medical-grade PU film to minimize sensor displacement relative to the body segment during swimming. The roll-angle signals derived from these two surface-mounted IMUs were used as estimates of upper-trunk and pelvic rotational behavior, respectively.

Data collection was conducted in a 50 m indoor swimming pool with a water depth ranging from 1.8 to 2.2 m. After a 30-min self-selected warm-up, swimmers completed a 400 m front crawl trial at maximal effort. Before each trial, participants stood quietly on land for approximately 5 s with their eyes facing forward and both feet aligned horizontally; the mean sensor orientation during this static standing period was used as the initial zero-offset reference, followed by Y-axis gravity-based rotation-matrix correction of the IMUs. The start was performed by pushing off from the pool wall rather than using a standard starting block. Performance level was evaluated relative to each swimmer’s season-best time. Completion times no more than 5% slower than each swimmer’s season-best time were considered acceptable (Psycharakis and Sanders, 2008). All swimmers met this criterion (international-level swimmers: 245.65 ± 2.34 s; national-level swimmers: 266.12 ± 7.99 s).

### 2.3 Data analysis

Following frequency-domain evaluation of the roll-angle and angular-velocity signals, all signals were smoothed using a fourth-order low-pass Butterworth filter with a 5 Hz cut-off (Averianova et al., 2016; Mooney et al., 2015). Turn phases were identified from the trunk mediolateral-axis (X-axis) angular velocity and excluded; analyses were restricted to straight-swimming segments between turns. A trunk roll cycle was defined as one complete bidirectional rotation of the roll angle about the body longitudinal axis (Y-axis) around the neutral position (0°), from one upward zero-crossing to the next upward zero-crossing.

Inter-segmental coupling was quantified using cross-correlation. Phase lag was defined as the lag at the peak correlation coefficient, normalized to roll-cycle duration, and expressed as % roll cycle (Nikodelis et al., 2005).

To provide an empirical basis for threshold selection, cycle-level phase-lag values were additionally examined using unsupervised K-means clustering (k = 3; Supplementary Figure S1; Supplementary Table S1). Based on the resulting cluster boundaries, phase lag < −1.5% was classified as an upper-trunk-leading pattern (UL), phase lag >1.5% as a pelvic-leading pattern (PL), and |phase lag| ≤ 1.5% as near-synchronous motion (NS). It should be noted that the cross-correlation lag provides a cycle-level summary of the dominant upper-trunk–pelvic lead–lag tendency and does not describe phase-specific changes, within-cycle reversals, or moment-to-moment variability in continuous coupling. Separately, as a supplementary methodological agreement check, Hilbert transform–derived mean relative phase was converted to a lag-equivalent % cycle measure and classified using the same threshold to evaluate agreement with the cross-correlation-based classification. Full details are provided in the Supplementary (Supplementary Tables S2, S3; Supplementary Figure S2).

Only cycles with a duration of 1.2–2.5 s and containing one major peak and one major trough in the upper-trunk-roll waveform were retained for further analysis. For each roll cycle, the following variables were computed:

- Torso twist range (TTR, °): the range of the instantaneous upper-trunk–pelvic roll difference within a roll cycle, calculated as the sum of the maximal left- and right-direction torso twist angles;
- Torso twist velocity (TTV, °/s): the mean absolute angular velocity of the upper-trunk–pelvic roll difference within a roll cycle;
- Upper-trunk roll range (UR, °) and pelvic roll range (PR, °): the range of upper-trunk roll and pelvic roll within a roll cycle, each calculated as the sum of the maximal left- and right-direction roll angles;
- Upper-trunk roll velocity (URV, °/s) and pelvic roll velocity (PRV, °/s): the mean absolute upper-trunk-roll and pelvic-roll angular velocities within a roll cycle, respectively.

### 2.4 Statistical analysis

Two Bayesian multilevel models were fitted. The primary analysis used a ±1.5% cross-correlation lag threshold to classify cycles, and sensitivity analyses were conducted with ±1% and ±2% thresholds to assess the robustness of the findings.

The first model compared the six kinematic variables across strategies: TTR, TTV, UR, PR, URV, and PRV. Each variable was z-standardized within athlete; therefore, the resulting strategy contrasts represent within-athlete relative differences and do not estimate absolute between-athlete differences in kinematic magnitude. A multivariate Bayesian multilevel Gaussian model was fitted with the six variables as simultaneous responses. Strategy was the main fixed effect, while 50-m segment, within-athlete speed variation, and between-athlete mean speed were included as covariates. The model included swimmer-level random intercepts and swimmer-level random slopes for strategy, allowing both baseline levels and strategy-related effects to vary across swimmers. Pairwise strategy differences were reported as posterior means and 95% credible intervals (95%CrI).

The second model examined the relative use of the three strategies across race segments and swimming speed. Cycle-level data were aggregated by swimmer and 50-m segment (S1–S8), yielding the counts of NS, UL, and PL cycles within each segment. A Bayesian multilevel multinomial logistic regression model was fitted, with the counts of the three strategies as the response and the total number of cycles in each segment specified as the multinomial trials. Fixed effects included 50-m segment, within-athlete speed variation, and between-athlete mean speed. The model included swimmer-level random intercepts and swimmer-level random slopes for 50-m segment, allowing both overall strategy preference and segment-related effects to vary across swimmers. Results were reported as odds ratios, 95% credible intervals (95%CrI), and model-based predicted probabilities.

Both models were fitted in R using the brms package (Bürkner, 2017). Four Markov chains were run, with 4,000 iterations per chain and 2000 warm-up iterations. Weakly informative priors were used: normal (0, 1) for fixed effects, normal (0, 1) or normal (0, 1.5) for intercepts, exponential (1) for random-effect standard deviations, and LKJ (2) for the residual correlation matrix in the multivariate model. Model convergence was assessed using Rhat, effective sample size, and divergent transitions (Kruschke, 2021).

## 3 Results

### 3.1 Overview of coordination patterns

Abbreviations: NS, near-synchronous; UL, upper-trunk-leading; PL, pelvic-leading. A1–A22 denote anonymized swimmer IDs.

Across the full 400 m, 2,796 valid roll cycles were included. NS was the most frequently observed strategy (1,483 cycles, 53.04%), followed by PL (723 cycles, 25.86%) and UL (590 cycles, 21.10%).

**FIGURE 2:**
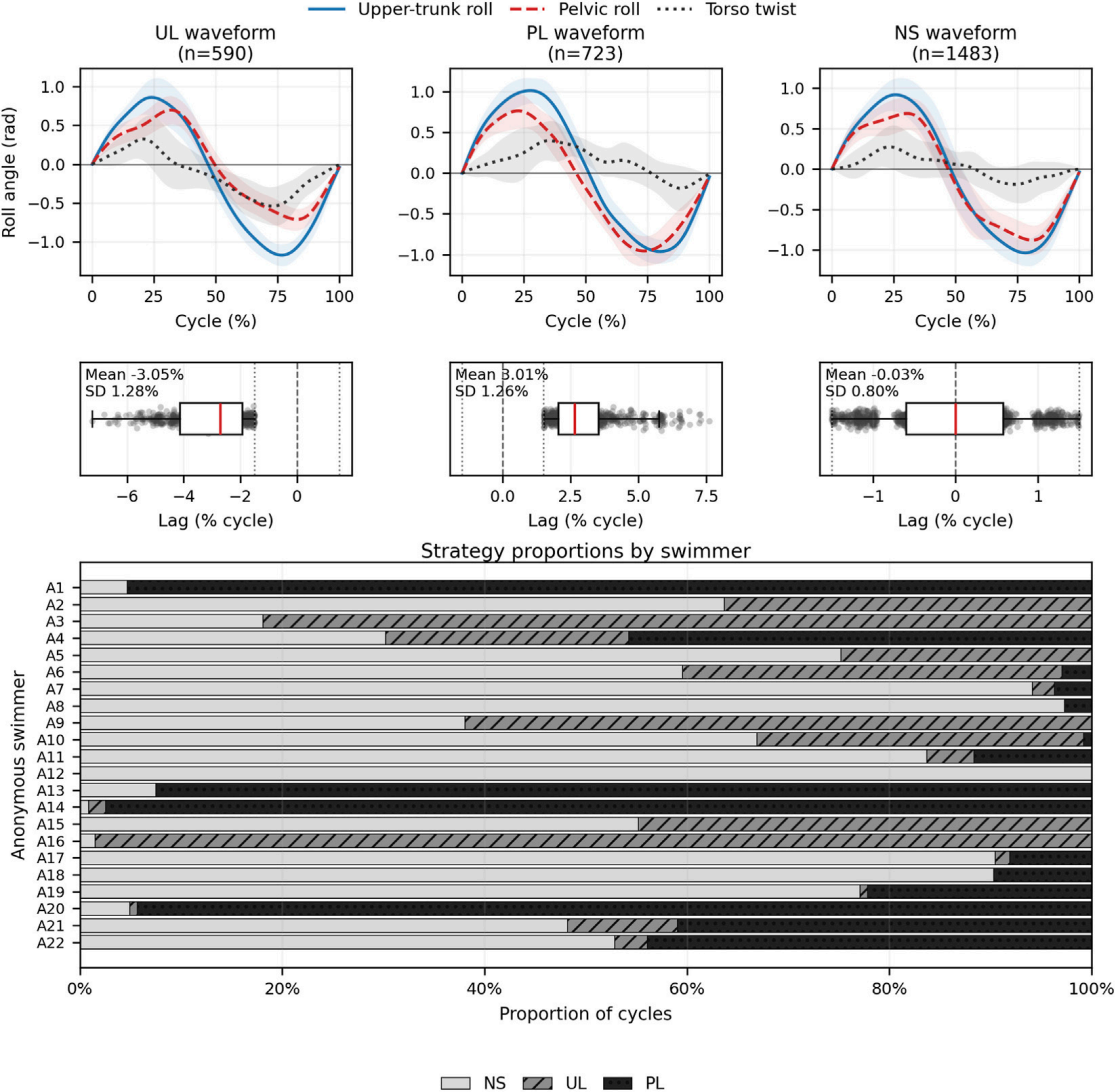
Mean roll-angle waveforms, cross-correlation lag distributions, and individual strategy proportions during the 400 m front crawl across upper-trunk–pelvic coordination strategies.

As shown in Figure 2, the upper-trunk and pelvic roll waveforms showed similar cyclic patterns across strategies, but differed in their temporal relationship. The upper trunk led the pelvis in UL (−3.05% ± 1.28%), the pelvis led the upper trunk in PL (3.01% ± 1.26%), and the two segments were closely aligned in NS (−0.03% ± 0.80%).

Strategy use varied substantially between swimmers. For each swimmer, the strategy with the highest proportion of valid cycles was identified as that swimmer’s predominant strategy. Based on this criterion, NS was the predominant strategy in 14 swimmers, PL in 5 swimmers, and UL in 3 swimmers. This indicates that swimmers differed mainly in their dominant strategy preference, although multiple strategies could still occur within the same swimmer.

Table 1 summarizes the strategy distribution and 50-m split times across the 400 m. NS was the most frequent strategy in all segments, ranging from 42.3% in S1 to 59.5% in S6. UL decreased from S1 to the middle segments and then increased again in the final segment, reaching 26.4% in S8. PL showed a comparatively smaller change across segments, ranging from 23.8% to 29.7%. The 50-m split times showed a U-shaped pattern, with faster times at the beginning and end of the race and slower times in the middle-to-late segments.

**TABLE 1:** Strategy distribution across 50-m segments during the 400 m front crawl.

Segment	NS	UL	PL	Pace (s)
S1	42.3%	28.1%	29.7%	29.19 ± 1.39
S2	54.0%	20.1%	25.9%	31.56 ± 1.40
S3	50.9%	20.1%	29.0%	32.62 ± 1.48
S4	55.5%	17.0%	27.5%	32.92 ± 1.41
S5	58.6%	17.6%	23.8%	33.39 ± 1.72
S6	59.5%	16.4%	24.0%	33.43 ± 1.91
S7	54.3%	21.4%	24.3%	33.68 ± 2.32
S8	49.8%	26.4%	23.8%	31.89 ± 2.34

Values represent the percentage of valid roll cycles classified into each strategy within each 50-m segment and 50-m segment pace time.

Descriptive statistics for the six kinematic variables are presented in Table 2. Across all three strategies, UR was consistently greater than PR. TTR was largest in UL and smallest in NS, while TTV and URV were also highest in UL. In contrast, PR and PRV were lowest in UL and higher in PL and NS.

**TABLE 2:** Descriptive statistics of kinematic variables across three upper-trunk–pelvic roll coordination strategies.

Variable	UL	PL	NS
TTR (°)	57.51 ± 19.15	48.09 ± 17.38	39.65 ± 16.04
TTV (°/s)	93.45 ± 40.56	71.52 ± 26.04	74.24 ± 42.60
UR (°)	120.03 ± 12.89	117.99 ± 12.34	117.61 ± 13.30
PR (°)	84.91 ± 11.57	104.18 ± 16.34	98.61 ± 13.55
URV (°/s)	150.48 ± 22.62	143.94 ± 27.86	141.53 ± 23.77
PRV (°/s)	100.70 ± 13.01	113.88 ± 19.31	110.97 ± 14.70

Data are presented as mean ± SD., Abbreviations; NS, near-synchronous; UL, upper-trunk-leading; PL, pelvic-leading; TTR, torso twist range; TTV, torso twist velocity; UR, upper-trunk roll range; PR, pelvic roll range; URV, upper-trunk roll velocity; PRV, pelvic roll velocity.

### 3.2 Kinematic differences among the three strategies

The multivariate Bayesian multilevel Gaussian model also showed acceptable convergence. The maximum Rhat was 1.007, the minimum bulk and tail effective sample sizes were 808.3 and 948.7, respectively, and no divergent transitions were observed.

Posterior pairwise contrasts for the six kinematic variables are presented in Table 3. TTR was higher in both UL and PL than in NS (NS - UL = −0.71, 95% CrI: 0.96, −0.42; NS - PL = −0.59, 95% CrI: 0.99, −0.20), while UL and PL did not differ clearly (UL - PL = 0.12, 95% CrI: 0.36, 0.57). For TTV, UL was higher than both NS and PL (NS - UL = −0.61, 95% CrI: 0.95, −0.26; UL - PL = 0.62, 95% CrI: 0.19, 1.04), whereas NS and PL were similar (NS - PL = 0.01, 95% CrI: 0.24, 0.25). For UR, PR, and PRV, NS and PL showed similar values, and both were higher than UL. Specifically, NS was higher than UL for UR (0.32, 95% CrI: 0.11, 0.57), PR (0.23, 95% CrI: 0.10, 0.38), and PRV (0.36, 95% CrI: 0.09, 0.66), while PL was also higher than UL for UR (UL - PL = −0.33, 95% CrI: 0.65, −0.02), PR (UL - PL = −0.26, 95% CrI: 0.47, −0.07), and PRV (UL - PL = −0.49, 95% CrI: 0.83, −0.18). URV did not show clear differences among strategies, with all 95% CrIs crossing 0.

**TABLE 3:** Posterior pairwise contrasts in kinematic variables across three trunk coordination strategies.

Variable	NS - UL	NS - PL	UL - PL
TTR	−0.71 [-0.96, −0.42]	−0.59 [-0.99, −0.20]	0.12 [-0.36, 0.57]
TTV	−0.61 [-0.95, −0.26]	0.01 [-0.24, 0.25]	0.62 [0.19, 1.04]
UR	0.32 [0.11, 0.57]	−0.01 [-0.24, 0.22]	−0.33 [-0.65, −0.02]
PR	0.23 [0.10, 0.38]	−0.04 [-0.21, 0.11]	−0.26 [-0.47, −0.07]
URV	−0.04 [-0.31, 0.26]	0.09 [-0.23, 0.41]	0.12 [-0.29, 0.54]
PRV	0.36 [0.09, 0.66]	−0.13 [-0.29, 0.03]	−0.49 [-0.83, −0.18]

Values are posterior mean differences (left - right) with 95% CrI. positive values favor the strategy on the left; negative values favor the strategy on the right. Intervals crossing 0 indicate no clear difference.

Sensitivity analyses using alternative ±1% and ±2% thresholds showed broadly consistent patterns, although effect sizes and their uncertainty varied across thresholds. The NS–UL contrasts were the most stable across thresholds, particularly for TTR, TTV, PR, and PRV, whereas comparisons involving PL were more sensitive to the classification threshold (Supplementary Table S4).

### 3.3 Changes in strategy probabilities across the 400-m split progression

Note: The dashed line at OR = 1 indicates no effect.

The Bayesian multilevel multinomial logistic regression model showed acceptable convergence. All Rhat values were approximately 1.00, the minimum bulk and tail effective sample sizes among the reported parameters were 1,677 and 2,900, respectively, and no divergent transitions were observed.

**FIGURE 3:**
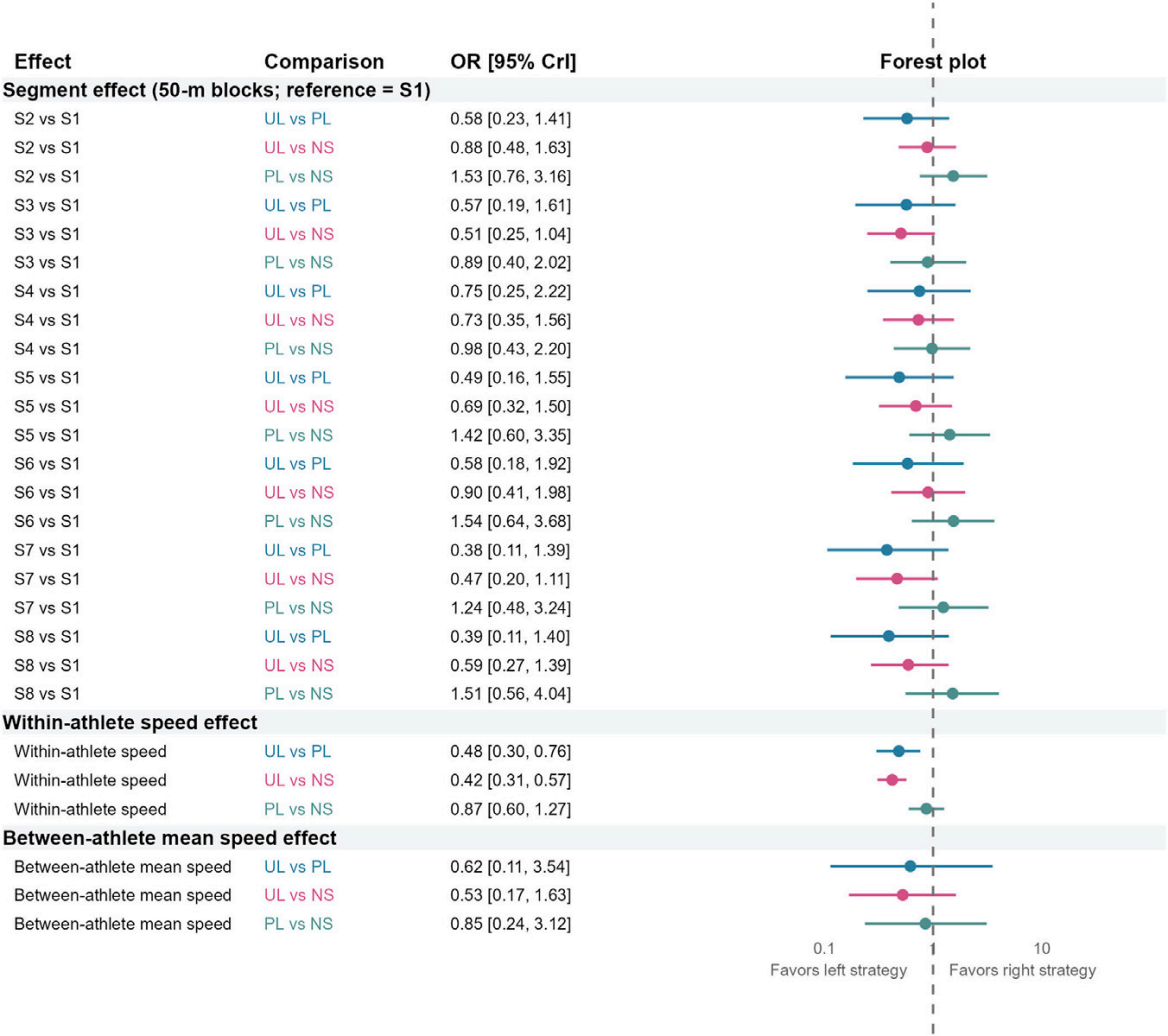
Effects of race segment and swimming speed on coordination strategy use in the Bayesian multinomial model.

Figure 3 presents the segment and speed effects from the Bayesian multilevel multinomial logistic regression model. No clear segment effects were observed for any strategy contrast, as all 95% credible intervals crossed 1. Although the point estimates for UL relative to NS and PL were higher in some later segments, these effects were not clearly supported by the posterior intervals.

For within-athlete speed, faster swimming was associated with higher odds of UL relative to both NS (OR = 2.38, 95% CrI: 1.76, 3.26) and PL (OR = 2.07, 95% CrI: 1.31, 3.33), whereas the PL versus NS contrast was not clearly affected by speed (OR = 1.15, 95% CrI: 0.79, 1.68). Between-athlete mean speed showed no clear effects, with all credible intervals crossing 1.

**FIGURE 4:**
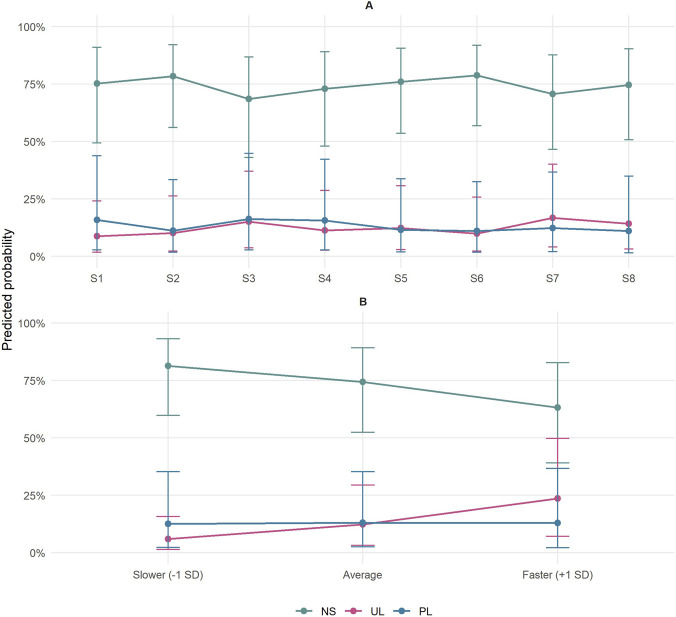
Model-based predicted probabilities of strategy use across race segments and within-athlete speed levels. **(A)** segment effect. **(B)** within-athlete speed effect.

As shown in Figure 4, across the 50-m segments, NS had the highest predicted probability in all segments (69%–79%). UL showed relatively low probabilities across segments (9%–17%), with slightly higher values in S3, S7, and S8. PL also remained relatively low across segments (11%–16%). For within-athlete speed, the predicted probability of UL increased from 6% at slower speed to 24% at faster speed, whereas NS decreased from 81% to 63%. PL showed little change across speed levels (13%–13%).

Sensitivity analyses using alternative thresholds showed generally consistent results. The within-athlete speed effects for UL versus NS and UL versus PL remained in the same direction across thresholds. PL versus NS was not clearly affected by speed at the ±1% and ±1.5% thresholds, but showed evidence of a positive speed effect at the ±2% threshold. No clear segment effects were consistently observed across thresholds (Supplementary Table S5).

## 4 Discussion

The present study investigated the occurrence patterns of three coordination strategies during body roll in front crawl swimming, their performance characteristics during the 400-m freestyle, and their associations with swimming performance. Three trunk coordination strategies were identified, namely, UL, PL, and NS, which corresponded to different temporal organizations of trunk segments. In addition, Figure 2 showed inter-individual differences in the use of trunk coordination strategies. This finding is largely consistent with previous studies (Psycharakis and Sanders, 2008; Sanders and Psycharakis, 2009; Psycharakis and McCabe, 2011). From the perspective of motor control, similar coordination outcomes can be achieved through different movement solutions. Swimmers may adopt different trunk coordination strategies to accomplish the same motor task according to their anthropometric characteristics, technical habits, and neuromuscular control capacities. This interpretation is consistent with the concept of degeneracy in motor control, whereby similar coordination outcomes can be achieved through different kinematic solutions (Seifert et al., 2016; Edelman and Gally, 2001).

One possible explanation for the presence of three coordination strategies is that the trunk may adjust its coordination pattern in response to different inter-limb coordination modes. Previous research has suggested that coordination among different trunk segments may help improve the efficiency of torque transfer from the trunk to the limbs (Dai et al., 2025). Therefore, swimmers may recruit trunk muscles to respond to, or anticipate, internal and external torques generated by upper- and lower-limb movements, thereby maintaining an optimal body position (Andersen et al., 2023). In front crawl swimming, shoulder rotation is closely related to arm movement (Yanai, 2003; Psycharakis and McCabe, 2011), whereas the timing of pelvic rotation is strongly synchronized with the kick action (Psycharakis and Ross, 2010). Therefore, the phase relationship between upper-trunk and pelvic rotation is likely to be influenced by inter-limb coordination. However, because the present study did not further measure kinematic parameters of the upper and lower limbs, this interpretation cannot yet be confirmed.

In terms of selection probability, NS was the most frequently used strategy during the 400-m freestyle. Regarding the speed effect, NS was more likely to be selected at the mean speed or below the mean speed, whereas its selection probability decreased sharply once swimming speed exceeded the mean. In terms of kinematic characteristics, UR and PR were significantly greater in NS than in UL, while they were similar to those observed in PL. This finding is consistent with previous research showing that swimmers exhibit greater body roll angles at lower swimming speeds (Yanai, 2003). The 400-m freestyle is a middle-distance event with a relatively long duration, requiring swimmers to avoid excessive energy expenditure during the initial phase while maintaining a relatively stable swimming speed during the middle portion of the race. Previous studies have shown that elite 400-m freestyle swimmers commonly adopt a fast-start-even or parabolic pacing pattern. Specifically, the initial phase is relatively fast because of the advantage provided by the start and push-off; speed then decreases and tends to stabilize over approximately the middle 300 m, followed by a certain degree of end-spurt in the final phase (Barroso et al., 2021; Mauger et al., 2012). In the present study, swimmers generally adopted a similar pacing pattern, which allowed swimming speed to remain relatively stable across several middle segments and may therefore explain the greater number of NS cycles observed during these segments.

Second, UL showed a relatively pronounced speed effect, indicating that swimmers were more likely to adopt UL as swimming speed increased. This finding is inconsistent with previous studies. Specifically, the studies by McCabe and Psycharakis found no significant correlations between swimming speed and the timing of the left or right peak or neutral positions of shoulder roll or hip roll, nor did they identify a specific phase-dominant pattern between shoulder and hip rotation (Psycharakis and McCabe, 2011; Psycharakis and Sanders, 2008). This discrepancy may be attributable to differences in measurement methods, testing protocols, and analytical approaches. Of the two studies by Psycharakis, one focused on 25-m swimming and the other focused on 200-m swimming, and both analyzed shoulder-hip rotation timing using a discrete-event-based method. In the 200-m study, only one cycle was selected from each 50-m segment, resulting in only four cycles across the entire trial. Although the three-dimensional video-based kinematic approach used in that study is a well-established method for quantifying swimming technique, its labor-intensive data collection procedures limited the number of stroke cycles that could be analyzed. Therefore, while this approach is suitable for describing representative mean movement patterns, it is less suited to evaluating cycle-to-cycle variability and transitions between coordination patterns. In the 25-m study, the within-athlete fluctuation in speed was relatively small, the number of analyzed cycles was also limited, and technical execution may have been more stable because of the short distance. These methodological differences may partly explain why previous studies did not report a significant association between shoulder–hip rotation timing and swimming speed. Meanwhile, UL showed greater TTR and TTV than NS, which is consistent with previous findings indicating that torso twist increases as swimming speed increases (Andersen et al., 2020; Hyodo et al., 2023).

The results for PL were relatively sensitive to the classification threshold, suggesting that PL may be a highly heterogeneous strategy. In terms of kinematic characteristics, PL also showed significantly greater TTR than NS, whereas UR and PR did not differ significantly from those in NS, and both were higher than those in UL. Psycharakis suggested that the timing of pelvic rotation is closely synchronized with the kick action (Psycharakis and Ross, 2010), which may provide a possible explanation for the high heterogeneity of PL. In front crawl coordination, kick actions are commonly classified as two-beat, four-beat, and six-beat patterns (Sortwell, 2011). As swimming speed increases, the proportion of six-beat kick use increases, and elite swimmers almost exclusively adopt the six-beat kick at higher speeds (Millet et al., 2002). Previous research has also indicated that, even within the same six-beat kick pattern, the rhythms of the trunk and lower limbs are not completely uniform but instead exhibit complex rhythmic organization (Sanders and Psycharakis, 2009). Therefore, the relatively high heterogeneity of PL does not undermine its explanatory value as an independent coordination strategy. Rather, it may indicate that PL is a coordination strategy that is more susceptible to the combined regulation of lower-limb rhythm, pelvic rotation timing, and speed context.

From the perspective of speed-related results, the association between PL and speed was weaker than that observed for UL, but it was not completely absent. In the sensitivity analysis of classification thresholds, PL showed a marginal speed effect relative to NS only under the 2% threshold, suggesting that PL may also occur in higher-speed contexts. This may partly explain why previous studies based on short-distance or sprint contexts often reported a group-level pattern in which the lower back, hip, or pelvis led the upper back or shoulder (Nikodelis et al., 2023; Lee et al., 2025). This may also suggest that the occurrence of PL is context dependent, and that PL may represent a coordination mode that is less frequently used during acceleration over longer distances. One possible explanation is that the leg kick in front crawl swimming is not the primary source of speed generation (Guignard et al., 2019), and that it has a relatively high metabolic cost (Morris et al., 2017). However, because the present study did not further measure energy expenditure or muscle activity characteristics under different strategies, this interpretation cannot yet be confirmed.

No significant segment effect was observed for the three strategies in the present study. In addition, the between-athlete mean speed effect was not significant, indicating that swimmers with higher overall swimming speeds were not more likely to adopt any particular strategy. Together with the individual differences in strategy preference shown in Figure 2, this finding further suggests that movement organization in skilled athletes does not necessarily converge toward a single standardized pattern. Instead, movement variability and inter-individual differences may reflect functional adaptations to individual, task, and environmental constraints (Seifert et al., 2014). Individualized coordination patterns may exist even under the same event and high-intensity conditions (Figueiredo et al., 2012). Therefore, the absence of segment-related differences in strategy use in the present study may reflect individualized strategy use by skilled swimmers in the context of the 400-m freestyle. This finding is consistent with McCabe and Ross, 2012, who found no significant differences in total shoulder and hip roll magnitude or in the timing of maximum shoulder and hip roll between sprint and distance specialists during a maximal 400-m front-crawl swim, despite differences in average swimming velocity. Together, these findings suggest that body-roll coordination during the 400-m freestyle may reflect individualized technical solutions rather than a single specialization- or speed-dependent pattern. Future studies should further examine strategy use at the individual-swimmer level in elite swimmers.

Several limitations of the present study should be acknowledged. First, only male swimmers were included; therefore, the generalizability of the findings, particularly to female swimmers, remains limited. Second, the present study used a single-trial design and did not assess the test-retest reliability of the strategy classification results across different testing days or repeated testing conditions. Therefore, the stability of different trunk coordination strategies across contexts requires further verification. Third, UL, PL, and NS were classified primarily on the basis of kinematic variables, and the classification results were influenced to some extent by the choice of threshold. Although the additional K-means analysis provided empirical support for the selected classification threshold, it should be interpreted as a threshold-supporting analysis rather than independent evidence that three coordination patterns naturally emerged from the data, because the number of clusters was predefined as k = 3. In addition, fatigue was not directly measured in the present study. Therefore, interpretations regarding the relationships between different strategies and fatigue accumulation or energy expenditure remain indirect. It should also be noted that the present study used IMUs to measure the rolling motion of the upper trunk and pelvis. Accordingly, the findings should be interpreted as a proxy representation of upper-trunk and pelvic roll coordination, and they cannot be considered fully equivalent to shoulder and hip joint or segment rotations measured using three-dimensional motion capture. Relatedly, no posture-specific prone calibration or synchronized three-dimensional motion-capture validation was performed. Therefore, the IMU-derived 0° roll reference should be interpreted as an operational kinematic reference for cycle segmentation rather than a directly measured anatomical neutral roll posture, which may influence side-specific half-cycle or absolute left–right angular measures. Finally, because both strategy classification and torso twist range were derived from upper-trunk and pelvic roll kinematics, there may be a certain degree of construct overlap or circularity between these variables. Future studies should integrate kinematic data with kinetic measurements, electromyographic data, and independent performance and fatigue indicators to further clarify the specific effects of each strategy and to develop individualized application schemes according to differences in sex, speed, fatigue level, and technical goals.

## 5 Conclusion

In conclusion, this study identified three trunk coordination strategies during the 400-m freestyle, namely, UL, PL, and NS, indicating that body roll in front crawl swimming has diverse and individualized characteristics. NS was the most common strategy and mainly occurred at mean or lower swimming speeds. UL was more likely to be adopted as swimming speed increased and showed greater torso twist range and torso twist velocity, suggesting that it may be associated with higher speed output. PL showed greater heterogeneity and may be more strongly regulated by lower-limb rhythm, pelvic rotation timing, and speed context. In addition, no significant segment effect or between-athlete mean speed effect was observed, suggesting that skilled swimmers may have individualized trunk coordination and strategy-use patterns. Future studies should integrate kinetic, electromyographic, and fatigue-related measures to further verify the functional significance of different strategies.

## Data Availability

The raw data supporting the conclusions of this article will be made available by the authors, without undue reservation.
