# Supplementary material for: Trunk rotation coordination in front crawl: which segment leads? Three coordination strategies and their dynamic changes across 400-m split progression in elite swimmers
Source: Front Bioeng Biotechnol. 2026 Jul 23;14:1857371. doi: 10.3389/fbioe.2026.1857371 (PMC13443057; doi:10.3389/fbioe.2026.1857371)
Supplement: Supplementary file 1 [file DataSheet1.pdf]

Supplementary Material

1 Results of K-means Clustering Analysis

Fig. S1. Threshold identification for cross-correlation lag using K-means clustering.

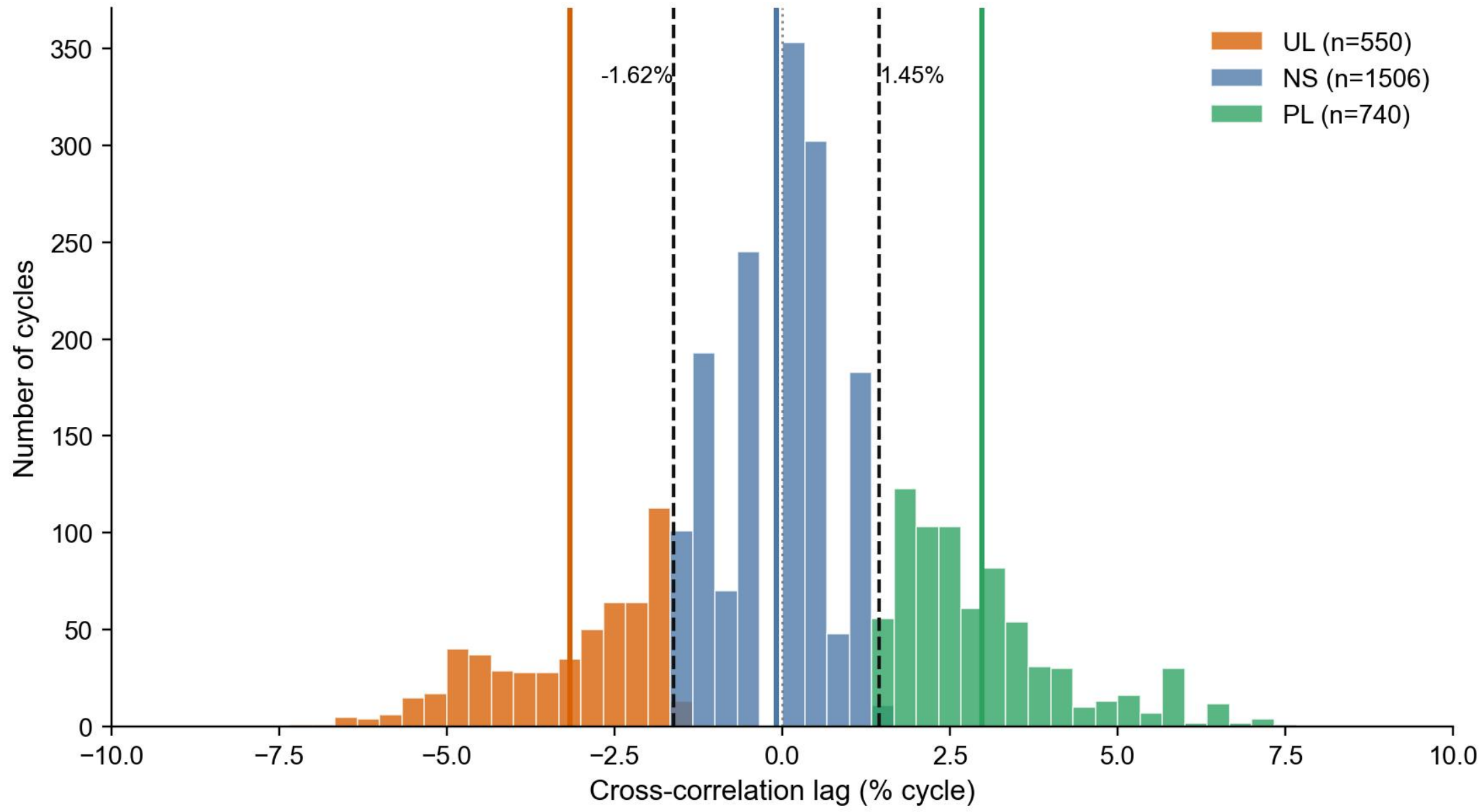

Table S1. Summary of K-means clustering results for cross-correlation lag.

K-means cluster	K-means class	center	n	mean	sd	min	max
1	UL	−3.16	550	−3.16	1.26	−7.24	−1.63
2	NS	−0.08	1506	−0.09	0.81	−1.61	1.43
3	PL	2.98	740	2.97	1.27	1.45	7.62

As shown in Table S1 and Fig. S1, one-dimensional K-means clustering of the cross-correlation lag identified three clusters, with centers at -3.16%, -0.08%, and 2.98% of the cycle, corresponding to UL, NS, and PL, respectively. The resulting cluster boundaries were -1.62% and 1.45% of the cycle. Based on this clustering, an approximately symmetric threshold of ±1.5% was adopted for the primary classification in the main analysis. Under this operational threshold, cycles with phase lag < -1.5%, > 1.5%, and within ±1.5% were classified as UL, PL, and NS, respectively. In the original K-means clustering result, NS was the largest cluster

(1506 cycles), followed by PL (740 cycles) and UL (550 cycles). The three clusters were clearly separated along the lag axis, with NS concentrated around zero lag and UL and PL distributed in the negative and positive lag regions, respectively.

2 Sensitivity Analysis of Hilbert Phase to Cross-Correlation Time Lag

A Hilbert-transform-based phase analysis was performed as a supplementary methodological check of the primary cross-correlation-derived lead–lag classification. The same preprocessed roll-angle signals and full-cycle segmentation used in the primary analysis were used for this analysis.

For each full roll cycle, the upper-trunk and pelvic roll-angle signals were first extracted from their corresponding cycle segments. The upper-trunk and pelvic roll-angle signals were then mean-centered within each cycle. For each signal, the analytic signal was obtained using the Hilbert transform, and the instantaneous phase was calculated from the angle of the analytic signal. The instantaneous upper-trunk and pelvic phases were unwrapped to avoid discontinuities caused by phase jumps. The continuous relative phase was calculated as:

$$\text{relative phase} = \text{upper-trunk phase} - \text{pelvic phase}$$

The relative phase was then wrapped to the $-\pi$ to π range using the circular representation of the phase difference. For each cycle, the circular mean and circular standard deviation of the relative phase were calculated. The circular mean relative phase was converted from radians to degrees.

To compare the Hilbert-derived phase measure with the cross-correlation lag, the mean relative phase was converted into a lag-equivalent cycle percentage:

$$\text{Hilbert lag-equivalent percentage} = -\text{ mean relative phase} / 360 \times 100$$

This sign convention was chosen to match the primary cross-correlation lag definition, where negative values indicate upper-trunk-leading timing and positive values indicate pelvic-leading timing. The Hilbert-derived lag-equivalent value was then classified using the same ±1.5% threshold used in the primary analysis: values within ±1.5% were classified as near-synchronous, values below −1.5% as upper-trunk-leading, and values above +1.5% as pelvic-leading.

Finally, the Hilbert-derived classification was compared with the cross-correlation-derived classification. Agreement was assessed at both the continuous level and the categorical level. Pearson and Spearman correlations were calculated between the cross-correlation lag percentage and the Hilbert-derived lag-equivalent percentage. Classification agreement was calculated as the proportion of cycles assigned to the same lead–lag category by the two methods, both overall and within each cross-correlation-based category.

Table S2. Overall agreement between cross-correlation lag and Hilbert-transform-derived lag-equivalent measures.

n	Pearson r	Spearman p	Overall classification agreement (%)	Mean absolute difference (% cycle)	Median absolute difference (% cycle)
2897	0.96	0.96	87.26	0.43	0.26

Table S3. Agreement by cross-correlation-based classification.

Cross-correlation classification	n	Cross-correlation lag (% cycle) Mean ± SD	Hilbert-equivalent lag (% cycle) Mean ± SD	Classification agreement (%)
PL	759	3.05±1.31	3.22±1.36	95.78
UL	602	-3.05±1.28	-3.04±1.54	86.21
NS	1536	-0.02±0.79	0.20±1.10	83.46

Figure S2. Agreement between cross-correlation lag and Hilbert-derived phase lag.

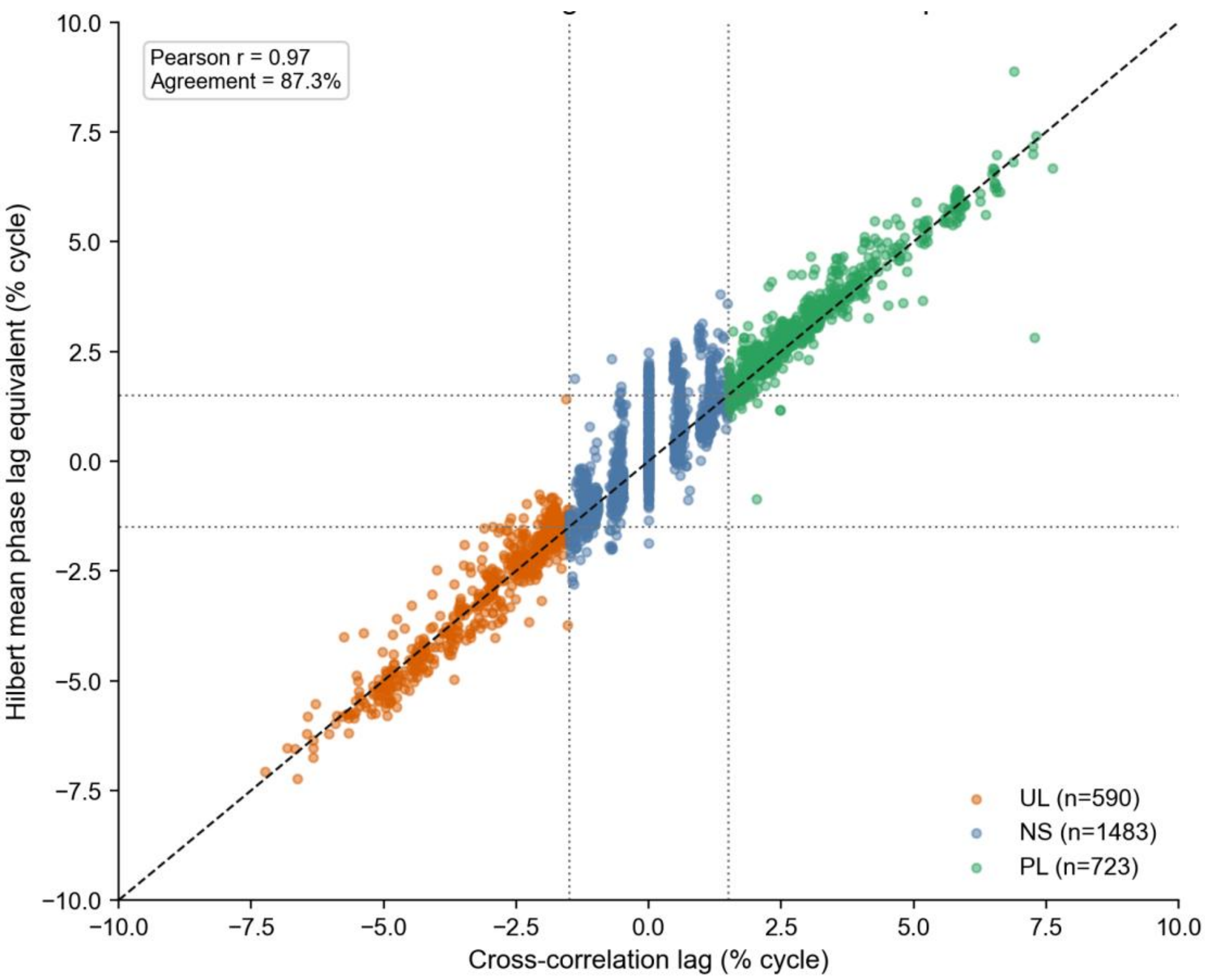

3 Threshold Sensitivity Analyses

3.1 Threshold Sensitivity of Kinematic Differences Among Strategies

Table S4. Threshold sensitivity analysis of strategy-related kinematic differences from the multivariate Bayesian multilevel Gaussian model

Outcome	Contrast	±1% posterior mean difference [95% CrI]	±1% conclusion	±1.5% posterior mean difference [95% CrI]	±1.5% conclusion	±2% posterior mean difference [95% CrI]	±2% conclusion
TTR	NS - UL	-0.53 [-0.76, -0.28]	Higher in UL	-0.71 [-0.96, -0.42]	Higher in UL	-0.88 [-1.17, -0.61]	Higher in UL
TTR	NS - PL	-0.27 [-0.58, 0.06]	Unclear	-0.59 [-0.99, -0.20]	Higher in PL	-0.92 [-1.64, -0.17]	Higher in PL
TTR	UL - PL	0.27 [-0.13, 0.66]	Unclear	0.12 [-0.36, 0.57]	Unclear	-0.04 [-0.82, 0.77]	Unclear
TTV	NS - UL	-0.50 [-0.78, -0.24]	Higher in UL	-0.61 [-0.95, -0.26]	Higher in UL	-0.85 [-1.30, -0.40]	Higher in UL
TTV	NS - PL	0.05 [-0.14, 0.23]	Unclear	0.01 [-0.24, 0.25]	Unclear	0.07 [-0.28, 0.42]	Unclear
TTV	UL - PL	0.55 [0.23, 0.86]	Higher in UL	0.62 [0.19, 1.04]	Higher in UL	0.92 [0.35, 1.49]	Higher in UL
UR	NS - UL	0.16 [-0.05, 0.36]	Unclear	0.32 [0.11, 0.57]	Higher in NS	0.36 [0.11, 0.62]	Higher in NS
UR	NS - PL	0.01 [-0.21, 0.22]	Unclear	-0.01 [-0.24, 0.22]	Unclear	0.07 [-0.68, 0.79]	Unclear
UR	UL - PL	-0.15 [-0.44, 0.12]	Unclear	-0.33 [-0.65, -0.02]	Higher in PL	-0.29 [-1.08, 0.48]	Unclear

PR	NS - UL	0.18 [0.05, 0.32]	Higher in NS	0.23 [0.10, 0.38]	Higher in NS	0.33 [0.14, 0.54]	Higher in NS
PR	NS - PL	-0.05 [-0.21, 0.07]	Unclear	-0.04 [-0.21, 0.11]	Unclear	0.21 [-0.15, 0.57]	Unclear
PR	UL - PL	-0.23 [-0.42, -0.07]	Higher in PL	-0.26 [-0.47, -0.07]	Higher in PL	-0.12 [-0.54, 0.29]	Unclear
URV	NS - UL	-0.05 [-0.30, 0.19]	Unclear	-0.04 [-0.31, 0.26]	Unclear	-0.04 [-0.31, 0.28]	Unclear
URV	NS - PL	-0.06 [-0.27, 0.17]	Unclear	0.09 [-0.23, 0.41]	Unclear	0.29 [-0.35, 0.96]	Unclear
URV	UL - PL	-0.01 [-0.32, 0.32]	Unclear	0.12 [-0.29, 0.54]	Unclear	0.33 [-0.38, 1.06]	Unclear
PRV	NS - UL	0.34 [-0.05, 0.69]	Unclear	0.36 [0.09, 0.66]	Higher in NS	0.49 [0.16, 0.84]	Higher in NS
PRV	NS - PL	-0.16 [-0.38, 0.12]	Unclear	-0.13 [-0.29, 0.03]	Unclear	0.25 [-0.19, 0.80]	Unclear
PRV	UL - PL	-0.50 [-0.90, -0.02]	Higher in PL	-0.49 [-0.83, -0.18]	Higher in PL	-0.23 [-0.80, 0.39]	Unclear

The multivariate Bayesian multilevel Gaussian models showed that some strategy-related kinematic differences were robust across classification thresholds. For TTR and TTV, the NS - UL contrasts were consistently negative across all three thresholds, with 95% CrIs excluding 0, indicating that UL showed higher TTR and TTV than NS. The UL - PL contrast for TTV was also consistently positive across thresholds, indicating higher TTV in UL than in PL. For PR, the NS - UL contrast consistently indicated higher values in NS than in UL across all thresholds. In contrast, some effects were more threshold-dependent. For example, the NS - PL contrast for TTR indicated higher values in PL than in NS at the ±1.5% and ±2% thresholds, but was unclear at the ±1% threshold. Similar threshold-dependent patterns were observed for UR, PRV, and several PL-related contrasts. URV showed no clear differences between strategies under any threshold. Overall, the sensitivity analysis indicates that the finding of higher TTR and TTV in UL relative to NS was the most robust, whereas some PL-related kinematic differences were more sensitive to the choice of classification threshold.

3.2 Threshold Sensitivity of Strategy Probability Changes Across the 400-m Split Progression

Table S5. Threshold sensitivity analysis of changes in strategy probabilities across the 400-m split progression.

Effect group	Effect	Comparison	±1% OR [95% CrI]	±1% conclusion	±1.5% OR [95% CrI]	±1.5% conclusion	±2% OR [95% CrI]	±2% conclusion
Segment effect	S2 vs S1	UL vs NS	0.97 [0.52, 1.81]	Unclear	1.13 [0.61, 2.08]	Unclear	1.14 [0.57, 2.26]	Unclear
Segment effect	S2 vs S1	PL vs NS	0.61 [0.32, 1.19]	Unclear	0.65 [0.32, 1.32]	Unclear	1.13 [0.55, 2.26]	Unclear
Segment effect	S2 vs S1	UL vs PL	1.58 [0.69, 3.67]	Unclear	1.74 [0.71, 4.39]	Unclear	1.01 [0.38, 2.67]	Unclear
Segment effect	S3 vs S1	UL vs NS	1.37 [0.67, 2.81]	Unclear	1.98 [0.96, 4.04]	Unclear	2.46 [1.13, 5.38]	Favors UL
Segment effect	S3 vs S1	PL vs NS	1.40 [0.69, 2.91]	Unclear	1.12 [0.50, 2.48]	Unclear	0.88 [0.37, 2.01]	Unclear
Segment effect	S3 vs S1	UL vs PL	0.97 [0.38, 2.60]	Unclear	1.76 [0.62, 5.21]	Unclear	2.78 [0.91, 8.37]	Unclear
Segment effect	S4 vs S1	UL vs NS	0.99 [0.48, 2.07]	Unclear	1.36 [0.64, 2.90]	Unclear	1.42 [0.62, 3.25]	Unclear
Segment effect	S4 vs S1	PL vs NS	0.78 [0.37, 1.64]	Unclear	1.02 [0.45, 2.30]	Unclear	1.97 [0.86, 4.33]	Unclear
Segment effect	S4 vs S1	UL vs PL	1.27 [0.46, 3.51]	Unclear	1.33 [0.45, 4.03]	Unclear	0.72 [0.23, 2.18]	Unclear
Segment effect	S5 vs S1	UL vs NS	1.06 [0.49, 2.25]	Unclear	1.44 [0.67, 3.15]	Unclear	1.19 [0.50, 2.78]	Unclear
Segment effect	S5 vs S1	PL vs NS	0.55 [0.24, 1.23]	Unclear	0.70 [0.30, 1.66]	Unclear	0.56 [0.23, 1.32]	Unclear

Effect group	Effect	Comparison	±1% OR [95% CrI]	±1% conclusion	±1.5% OR [95% CrI]	±1.5% conclusion	±2% OR [95% CrI]	±2% conclusion
Segment effect	S5 vs S1	UL vs PL	1.93 [0.66, 5.71]	Unclear	2.05 [0.65, 6.44]	Unclear	2.13 [0.64, 6.95]	Unclear
Segment effect	S6 vs S1	UL vs NS	1.16 [0.52, 2.48]	Unclear	1.11 [0.51, 2.43]	Unclear	1.09 [0.46, 2.60]	Unclear
Segment effect	S6 vs S1	PL vs NS	0.56 [0.24, 1.32]	Unclear	0.65 [0.27, 1.57]	Unclear	0.86 [0.35, 2.05]	Unclear
Segment effect	S6 vs S1	UL vs PL	2.05 [0.68, 6.23]	Unclear	1.72 [0.52, 5.50]	Unclear	1.27 [0.38, 4.27]	Unclear
Segment effect	S7 vs S1	UL vs NS	1.83 [0.78, 4.24]	Unclear	2.14 [0.90, 5.12]	Unclear	2.04 [0.80, 5.15]	Unclear
Segment effect	S7 vs S1	PL vs NS	0.85 [0.34, 2.12]	Unclear	0.80 [0.31, 2.07]	Unclear	0.66 [0.26, 1.67]	Unclear
Segment effect	S7 vs S1	UL vs PL	2.16 [0.64, 7.34]	Unclear	2.66 [0.72, 9.44]	Unclear	3.08 [0.84, 11.23]	Unclear
Segment effect	S8 vs S1	UL vs NS	1.65 [0.75, 3.49]	Unclear	1.69 [0.72, 3.74]	Unclear	2.24 [0.87, 5.42]	Unclear
Segment effect	S8 vs S1	PL vs NS	0.83 [0.34, 2.16]	Unclear	0.66 [0.25, 1.79]	Unclear	0.60 [0.25, 1.47]	Unclear
Segment effect	S8 vs S1	UL vs PL	1.99 [0.62, 6.32]	Unclear	2.56 [0.72, 8.79]	Unclear	3.73 [1.04, 13.05]	Favors UL
WAS effect	WAS	UL vs NS	1.93 [1.43, 2.61]	Favors UL	2.38 [1.76, 3.26]	Favors UL	2.60 [1.84, 3.72]	Favors UL
WAS effect	WAS	PL vs NS	1.03 [0.73, 1.45]	Unclear	1.15 [0.79, 1.68]	Unclear	1.42 [1.00, 2.02]	Borderline favors PL
WAS effect	WAS	UL vs PL	1.87 [1.22, 2.90]	Favors UL	2.07 [1.31, 3.33]	Favors UL	1.83 [1.13, 3.00]	Favors UL

Effect group	Effect	Comparison	±1% OR [95% CrI]	±1% conclusion	±1.5% OR [95% CrI]	±1.5% conclusion	±2% OR [95% CrI]	±2% conclusion
BAS effect	BAS	UL vs NS	1.97 [0.76, 4.98]	Unclear	1.90 [0.62, 5.96]	Unclear	2.05 [0.63, 6.61]	Unclear
BAS effect	BAS	PL vs NS	0.87 [0.27, 2.96]	Unclear	1.18 [0.32, 4.24]	Unclear	1.06 [0.28, 3.95]	Unclear
BAS effect	BAS	UL vs PL	2.25 [0.49, 10.04]	Unclear	1.62 [0.28, 8.83]	Unclear	1.93 [0.33, 10.89]	Unclear

Note: **WAS**, within-athlete speed; **BAS**, between-athlete mean speed.

Fig. S3. Threshold sensitivity analysis of model-based predicted probabilities of strategy use across race segments and within-athlete speed levels.

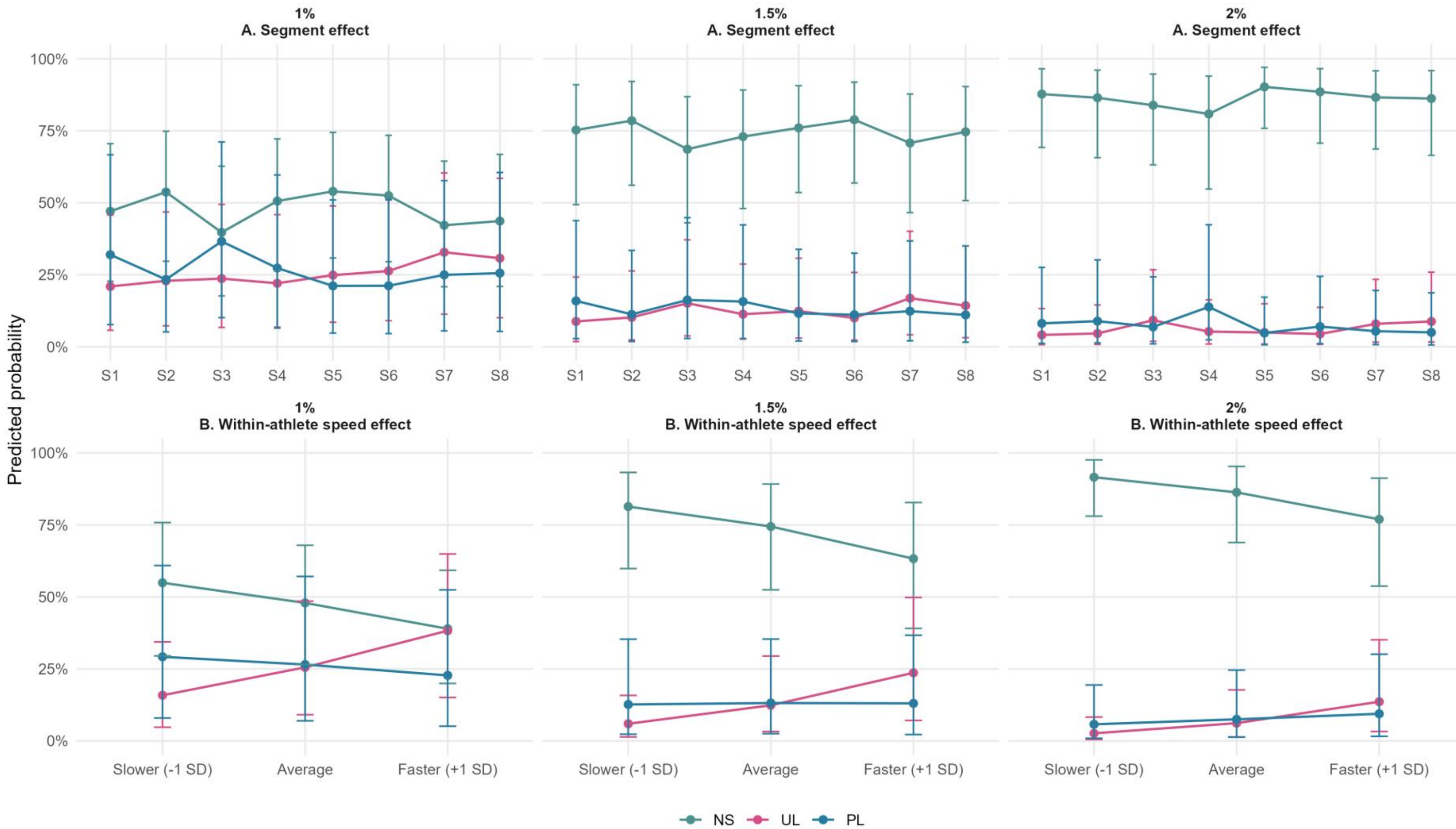

The Bayesian multilevel multinomial logistic regression models showed broadly consistent results across classification thresholds. The within-athlete speed effect showed the most stable pattern: faster swimming was associated with higher odds of UL relative to NS (±1%: OR = 1.93, 95% CrI: 1.43, 2.61; ±1.5%: OR = 2.38, 95% CrI: 1.76, 3.26; ±2%: OR = 2.60, 95% CrI: 1.84, 3.72) and higher odds of UL relative to PL (±1%: OR = 1.87, 95% CrI: 1.22, 2.90; ±1.5%: OR = 2.07, 95% CrI: 1.31, 3.33; ±2%: OR = 1.83, 95% CrI: 1.13, 3.00). The speed effect for PL relative to NS was unclear at the ±1% and ±1.5% thresholds, but showed a borderline trend at the ±2% threshold (OR = 1.42,

95% CrI: 1.00, 2.02). Segment effects were more threshold-sensitive: most segment contrasts remained unclear across all thresholds, with only a small number of contrasts showing a clear direction under a single threshold. Overall, the sensitivity analysis indicates that the association between higher within-athlete speed and greater UL use was robust, whereas segment effects and PL-related effects were more sensitive to threshold choice

4 Exploratory Level-Group Analyses

4.1 Exploratory Level Differences in Strategy Probabilities

Table S6. Exploratory international-national level differences in strategy probabilities across the 400-m split progression.

Level contrast	Strategy contrast	log-odds difference [95% CrI]	OR [95% CrI]	Posterior Pr(diff > 0)	Conclusion
international - national	UL vs NS	-0.03 [-1.80, 1.68]	0.97 [0.16, 5.36]	0.487	95% CrI crosses 1
international - national	PL vs NS	0.27 [-1.52, 2.10]	1.30 [0.22, 8.15]	0.611	95% CrI crosses 1
international - national	UL vs PL	-0.30 [-2.85, 2.17]	0.74 [0.06, 8.78]	0.414	95% CrI crosses 1

The exploratory level comparison showed no clear differences in strategy probabilities between international- and national-level swimmers. For UL relative to NS, the OR for international versus national swimmers was 0.97 (95% CrI: 0.16, 5.36). For PL relative to NS, the OR was 1.30 (95% CrI: 0.22, 8.15). For UL relative to PL, the OR was 0.74 (95% CrI: 0.06, 8.78). The 95% credible intervals crossed 1 for all three comparisons, and the posterior directional probabilities did not indicate a clear direction. Thus, this exploratory analysis did not provide evidence for systematic level-related differences in strategy use.

4.2 Exploratory Level Differences in Strategy-Specific Kinematic Variables

Table S7. Exploratory international-national level differences in strategy-specific kinematic variables from the multivariate Bayesian multilevel Gaussian model.

Outcome	Strategy	Level contrast	Posterior mean difference [95% CrI]	Posterior Pr(diff > 0)	Conclusion
TTR	NS	international - national	0.15 [-0.43, 0.72]	0.705	95% CrI crosses 0
TTR	UL	international - national	0.45 [-0.33, 1.26]	0.881	95% CrI crosses 0
TTR	PL	international - national	-0.30 [-1.18, 0.60]	0.247	95% CrI crosses 0
TTV	NS	international - national	0.33 [-0.07, 0.78]	0.944	95% CrI crosses 0
TTV	UL	international - national	0.63 [-0.09, 1.41]	0.959	95% CrI crosses 0
TTV	PL	international - national	0.00 [-0.56, 0.62]	0.489	95% CrI crosses 0
UR	NS	international - national	-0.07 [-0.34, 0.21]	0.298	95% CrI crosses 0
UR	UL	international - national	-0.12 [-0.59, 0.36]	0.306	95% CrI crosses 0
UR	PL	international - national	0.08 [-0.43, 0.55]	0.648	95% CrI crosses 0
PR	NS	international - national	-0.10 [-0.32, 0.10]	0.169	95% CrI crosses 0
PR	UL	international - national	-0.14 [-0.43, 0.18]	0.156	95% CrI crosses 0

Outcome	Strategy	Level contrast	Posterior mean difference [95% CrI]	Posterior Pr(diff > 0)	Conclusion
PR	PL	international - national	0.17 [-0.21, 0.55]	0.828	95% CrI crosses 0
URV	NS	international - national	0.16 [-0.15, 0.48]	0.845	95% CrI crosses 0
URV	UL	international - national	-0.19 [-0.81, 0.39]	0.262	95% CrI crosses 0
URV	PL	international - national	-0.02 [-0.70, 0.68]	0.476	95% CrI crosses 0
PRV	NS	international - national	0.07 [-0.20, 0.37]	0.677	95% CrI crosses 0
PRV	UL	international - national	-0.48 [-1.04, 0.07]	0.043	95% CrI crosses 0
PRV	PL	international - national	0.33 [-0.01, 0.74]	0.970	95% CrI crosses 0

The exploratory level comparison showed no clear differences in the six strategy-specific kinematic variables between international- and national-level swimmers. Across TTR, TTV, UR, PR, URV, and PRV, the 95% credible intervals for the international-national posterior mean differences crossed 0 under all three strategies. Some comparisons showed directional tendencies, such as higher TTV under UL in international swimmers (0.63, 95% CrI: -0.09, 1.41) and higher PRV under PL (0.33, 95% CrI: -0.01, 0.74), but these intervals still included 0. Therefore, this exploratory analysis did not provide evidence for systematic level-related differences in strategy-specific kinematic characteristics.
